# Author Correction: Integrated molecular subtyping defines a curable oligometastatic state in colorectal liver metastasis

**DOI:** 10.1038/s41467-018-07303-w

**Published:** 2018-11-13

**Authors:** Sean P. Pitroda, Nikolai N. Khodarev, Lei Huang, Abhineet Uppal, Sean C. Wightman, Sabha Ganai, Nora Joseph, Jason Pitt, Miguel Brown, Martin Forde, Kathy Mangold, Lai Xue, Christopher Weber, Jeremy P. Segal, Sabah Kadri, Melinda E. Stack, Sajid Khan, Philip Paty, Karen Kaul, Jorge Andrade, Kevin P. White, Mark Talamonti, Mitchell C. Posner, Samuel Hellman, Ralph R. Weichselbaum

**Affiliations:** 1 Department of Radiation and Cellular Oncology, The University of Chicago, Chicago, IL 60637 USA; 2 Ludwig Center for Metastasis Research, The University of Chicago, Chicago, IL 60637 USA; 3 Center for Research Informatics, The University of Chicago, Chicago, IL 60637 USA; 4 Department of Surgery, The University of Chicago, Chicago, IL 60637 USA; 5 Department of Surgery, Southern Illinois University, Springfield, IL 62702 USA; 6 Department of Pathology, NorthShore University Hospital, Evanston, IL 60201 USA; 7 Institute for Genomics and Systems Biology, The University of Chicago, Chicago, IL 60637 USA; 8 Department of Pathology, The University of Chicago, Chicago, IL 60637 USA; 9 Department of Surgery, Yale School of Medicine, New Haven, CT 06510 USA; 10 Department of Surgery, Memorial Sloan-Kettering Cancer Center, New York, NY 10065 USA; 11 Tempus Labs, Chicago, IL 60654 USA; 12 Department of Surgery, NorthShore University Hospital, Evanston, IL 60201 USA

Correction to: *Nature Communications*; 10.1038/s41467-018-04278-6, published online 04 May 2018.

In the originally published version of this Article, the affiliation details for Kevin P. White inadvertently omitted ‘Tempus Labs, Chicago, IL, 60654, USA’. This has now been corrected in both the PDF and HTML versions of the Article.

