# Supplementary material for: Integrated molecular subtyping defines a curable oligometastatic state in colorectal liver metastasis
Source: Nat Commun. 2018 May 4;9:1793. doi: 10.1038/s41467-018-04278-6 (PMC5935683; doi:10.1038/s41467-018-04278-6)
Supplement: Supplementary file 1 — Supplementary Information [file 41467_2018_4278_MOESM1_ESM.pdf]

Pitroda et al: Integrated Molecular Subtyping Defines A Curable Oligometastatic State in Colorectal Liver Metastasis

Supplementary Information for: "Integrated Molecular Subtyping Defines A Curable Oligometastatic State in Colorectal Liver Metastasis"

<u>Table of Contents</u>

A. Supplementary Methods (pp. 3-15)

1. Clinical Information (pp. 3-4)
2. Nucleic Acid Extraction (p. 4)
3. RNA Sequencing (pp. 4-5)
4. microRNA Expression Profiling (pp. 5-6)
5. Consensus Clustering of Expression Data (p. 6-7)
6. Consensus Molecular Subtyping (CMS) of Colorectal Liver Metastases (p. 7-8)
7. Similarity Network Fusion (pp. 8-9)
8. Ensemble of Gene Set Enrichment Analyses (EGSEA) (p. 9-10)
9. SNF Class Predictor (pp. 10-11)
10. Hybrid Capture Next Generation Sequencing (pp. 11-14)
11. Microsatellite Instability (MSI) Analysis (pp. 14)
12. Immunohistochemical Analysis (pp. 14-15)

B. Supplementary Figures and Figure Legends (pp. 16-31)

1. Supplementary Figure 1: Overview of study design. (p. 16)
2. Supplementary Figure 2: Overall survival by Consensus Molecular Subtypes (CMS) in patients with colorectal liver metastases. (p. 17)
3. Supplementary Figure 3: Consensus clustering analysis of the mRNA expression data for 95 patients with colorectal liver metastases. (pp. 18)
4. Supplementary Figure 4: Consensus clustering analysis of the miRNA expression data for 116 patients with colorectal liver metastases. (pp. 19)
5. Supplementary Figure 5: Median Silhouette Index (SI) for the SNF clusters under 72 parameter settings. (p. 20)

6. Supplementary Figure 6: Associations of molecular subtypes and clinicopathological variables. (p. 21)
7. Supplementary Figure 7: Non-random association of SNF network structure with overall survival in metastatic colorectal cancer patients. (p. 22)
8. Supplementary Figure 8: Distant metastasis-free survival by molecular subtype. (p. 23)
9. Supplementary Figure 9: Primary CRC CMS subtype by metastasis subtype. (p. 24)
10. Supplementary Figure 10: Perioperative chemotherapy regimens and associations with molecular subtypes. (p. 25)
11. Supplementary Figure 11: Prediction Analysis of Microarrays (PAM)-based classifier to distinguish molecular subtypes. (p. 26)
12. Supplementary Figure 12: Histologic analysis by molecular subtypes of liver metastasis. (p. 27)
13. Supplementary Figure 13: Oncoprint plot of exomic mutations occurring in 59 patients with colorectal liver metastases. (p. 28)
14. Supplementary Figure 14: Cytotoxic immune signature by molecular subtypes. (p. 29)
15. Supplementary Figure 15: Overall survival by integration of molecular subtype and Clinical Risk Scores (CRS). (p. 30)
16. Supplementary Figure 16: Metastatic recurrence patterns by integrated risk classification. (p. 31)

C. Supplementary References (p. 32-33)

A. Supplementary Methods

1. Clinical Information

Patient Characteristics

We conducted a retrospective clinical cohort study of patients who underwent hepatic resection of histologically confirmed metastatic colorectal adenocarcinoma at the University of Chicago Medical Center (Chicago, IL) and NorthShore University Health System (Evanston, IL) between 1994 and 2012. During this time period, approximately 60-75 patients per year underwent hepatic resection of colorectal liver metastases at the two participating institutions. All available clinical, pathologic, radiologic, and outcome data were collected for patients using medical records. Patients with unresectable or extrahepatic disease at the time of metastatic diagnosis were excluded from this study. In total, 134 consecutive patients with metastatic colorectal cancer who underwent surgical resection of limited *de novo* liver metastases were selected for molecular analysis. Patients were uniformly treated with perioperative chemotherapy, definitive treatment of primary colorectal cancer, and partial hepatectomy for resection of liver metastases. Detailed cohort characteristics are provided in **Table 1** and **Supplementary Table 1**. This study was approved by the Institutional Review Boards at each respective institution. Consent was waived for all enrolled subjects as this study was purely retrospective. Dates of recurrence, death or last follow-up were extracted from medical records and Social Security Death databases. Clinical risk scores (CRS) were calculated as previously described[1].

Pathologic Examination

Formalin-fixed paraffin-embedded (FFPE) specimens were collected from archived pathologic tissue. FFPE specimens were catalogued and histologically reviewed by an expert pathologist (Dr. Nora Joseph) to ensure adequacy of the specimen and histologic quality control. Tissue blocks containing sufficient tumor tissue were subjected to 2mm punch biopsies of both tumor and normal liver regions. For each surgical specimen, representative FFPE tissue blocks and corresponding H&E slides were analyzed to confirm the diagnosis of colorectal adenocarcinoma and identify regions containing high quantities of viable tumor cells, as well as independent regions containing normal liver parenchyma. Three cores from tumor and normal tissue regions were obtained. For each specimen, all three cores were combined to

reduce intratumoral variability. This procedure was repeated for both tumor and normal biopsies for each patient.

2. Nucleic Acid Extraction

Punch biopsy specimens were deparaffinized and processed using the RecoverAll Total Nucleic Acid Isolation Kit (Ambion, TX) according to the manufacturer's instructions. Briefly, 200μL of digestion buffer and 4μL of protease were added to each sample and incubated overnight at 55°C. RNA and DNA were extracted following the RecoverAll protocol according to the manufacturer's recommendations. Nucleic acid quantification was performed using a NanoDrop 1000 Spectrophotometer and a Qubit® Fluorometer. Nucleic acid extracts were stored at -80°C until further analysis.

3. RNA Sequencing

Library Construction

RNA integrity and quantity were evaluated using an Agilent 2100 Bioanalyzer (Agilent Technologies, CA). Reverse-stranded paired-end 75 base-pair sequencing libraries were constructed using Illumina Total RNA Stranded Kits. Ribosomal RNAs (rRNAs) were depleted by using the Ribo-Zero rRNA Removal Kit (Illumina). Libraries were sequenced on a HiSEQ2500 machine using standard reagents and protocols provided by Illumina. In total, 95 metastatic samples were successfully sequenced using this approach.

Read Alignment and Quantification

Unless otherwise specified, all data analyses were performed under the R programming and software environment for statistical computing and graphics version 3.3 (R Core Team, 2016). FastQ files for each sample were assessed for quality using the FastQC tool (version 0.11.2). Raw reads were aligned to the GRCh38 primary genome assembly using Spliced Transcripts Alignment to a Reference (STAR) aligner (version 2.4.2a) 1-pass algorithm[2]. After sorting the bam files in lexicographical order with the sambamba program[3], we assigned the reads to exon features annotated in GENCODE (release 22) using the FeatureCounts tool from the subread package (version 1.4.6) and summarized the read counts by genes[4]. The post-alignment quality control was carried out with Picard tools (version 1.117) and RSeQC

package (version 2.3.1). Specifically, we examined the QC data regarding the alignment summary, gene body coverage, read distribution, and ribosomal RNA depletion rate.

Data Normalization

We used functions in the R/Bioconductor package edgeR to extract the raw counts of the reads that were mapped to the protein-coding genes[5]. After removing the genes with zero read counts across all samples, we calculated the normalization factors to scale the raw library sizes and the log2-transformed count per million (log-CPM) for the expression level of each gene. The log-CPM values were corrected for batch effect (sequencing lane effect and institution) using removeBatchEffect function from the R/Bioconductor package limma[6]. We retained 18,714 genes for the subsequent analyses.

Detection of Differentially Expressed mRNAs

To identify differentially expressed mRNAs among samples grouped by Similarity Network Fusion (SNF – see **Section 7**) clusters, we first removed non/low-expressed genes in comparison groups by requiring read counts to be at least 1 across a minimum number of samples in one of the comparison groups, followed by trimmed mean of M-values (TMM) normalization using the calcNormFactors function in the edgeR package. Next, we removed heteroscedascity from the count data using the voomWithQualityWeights function from the limma package with quantile normalization method enabled. We then fit a linear model for each gene using the limma algorithm, adjusted for batch effect, and ranked the genes for differential expression using the empirical Bayes method with trend and robust options enabled. The differentially expressed genes were identified with the Benjamini–Hochberg procedure for multiple test adjustment and fold-change. The adjusted P-value threshold and fold-change threshold were set at 0.05 and 2.0, respectively (**Supplementary Table 2**).

4. microRNA Expression Profiling

RNA integrity and quantity were evaluated using an Agilent 2100 Bioanalyzer (Agilent Technologies, CA). Total RNA (500ng) was processed for biotin labeling according to the Affymetrix Flash Tag Biotin HSR RNA labeling guide (Affymetrix, CA). The biotin-labeled target was hybridized to Affymetrix miRNA 4.0 Array Chips for 16h at 48°C and 60rpm in an Affymetrix 640 hybridization oven. Arrays were washed and stained in an Affymetrix Fluidics Station 450 according to the Affymetrix GeneChip expression guide. The

arrays were scanned using the Affymetrix GeneChip Scanner 3000 7G. CEL intensity files were generated using GCOS software. In total, 116 metastatic samples were successfully assayed using this approach.

Data Pre-Processing and Normalization

The methods used in this analysis are available as part of the R/Bioconductor packages affy, oligo, limma and sva[6,7]. The raw Affymetrix GeneChip miRNA 4.0 Array CEL files were imported to R using the read.celfiles function from the oligo package. We first performed robust normexp-by-control background correction using the nec function from the limma package with the robust option enabled[8]. We then normalized the log2-transformed expression data using cyclic loess normalization with the array weight method. Finally, we summarized the probes into probesets using the rma function from the affy package with the options normalize and background disabled. To remove batch effects caused by array processing dates and the patient cohorts, we applied the ComBat algorithm implemented in the sva package[9]. We considered two batch factors: (1) institution and (2) microarray scan date. A single sample was run in batch 6 and combined with samples from batch 5. We removed the non/low-expressed probesets and retained the probesets representing 778 mature human miRNAs for the subsequent analyses.

Detection of Differentially Expressed miRNAs

We applied the limma method to identify differentially expressed miRNAs among the samples grouped by SNF clusters. We first estimated the relative quality weights for each array using the arrayWeightsSimple function, and then fit a linear model for each probeset adjusted for batch effect, followed by ranking probesets for differential expression using empirical Bayes method. The differentially expressed miRNAs were identified with the Benjamini–Hochberg procedure for multiple test adjustment and fold-change. The adjusted P-value threshold and fold-change threshold were set at 0.05 and 2.0, respectively (**Supplementary Table 3**).

5. Consensus Clustering of Expression Data

We performed unsupervised consensus clustering analysis on independent mRNA and miRNA expression data sets using the R package ConsensusClusterPlus (version 1.38.0). We selected the most

informative mRNAs or miRNAs for clustering, which consisted of the top 25% most variable mRNAs or miRNAs, as measured by the median absolute deviation (MAD). Normalized expression data from previous procedures were first standardized using the data normalization function in the R package clusterSim (version 0.45-1). To run ConsensusClusterPlus, we preset the options as a maximum evaluated cluster k = 6, 80% samples per resampling, 1,000 resamplings, Euclidean distance, and k-means clustering algorithm. We chose complete and average linkage as the inner-linkage and final linkage, respectively. The optimal number of k clusters was inferred by inspecting the consensus cumulative distribution function (CDF) plot and the proportion of ambiguously clustered pairs (PAC) plot where the optimal k corresponds to the lowest PAC[10] (k = 2, **Supplementary Fig. 3** and **Supplementary Fig. 4**).

6. Consensus Molecular Subtyping (CMS) of Colorectal Liver Metastases

Microarray expression data derived from 183 patients with colorectal liver metastasis were collected from ArrayExpress (study IDs: E-MTAB-1951 [MSK2], E-GEOD-62322 [French], E-GEOD-41258 [MSK3], and E-GEOD-35834 [Italian]). Study E-MTAB-1951 contains 96 samples profiled on the Illumina HumanHT-12 v3.0 Expression BeadChip. E-GEOD-62322 and E-GEOD-41258 contain 19 and 47 samples that were profiled on Affymetrix HG-U133A Arrays, respectively. E-GEOD-35834 consists of 27 samples profiled on the Affymetrix Human Exon 1.0 ST Array. We also used two sets of normalized RNA Sequencing data. One cohort includes 93 metastases from our cohort which were reanalyzed with RSEM to assess TPM abundances, while the other cohort contains 45 unpublished liver metastases that were obtained from the Memorial Sloan-Kettering Cancer Center from Dr. Sajid Khan and Dr. Philip Paty [MSK1] and processed similar to previously described methods in **Section 3**. For E-MTAB-1951, raw expression data was preprocessed with variance stabilizing transformation and quantile normalization using the lumi package (version 2.26.3). For the remaining microarray studies, CEL files were downloaded directly from ArrayExpress and processed with fRMA (version 1.28.0) for core annotation targets summarized by robust weighted average. Level 3 TCGA READ and COAD RNA Sequencing RSEM expression data was obtained from Sage-Bionetworks Synapse repository (syn: syn2320098, syn2320092, syn2320147, and syn2320079). TPM expression data corresponding to primary tumor samples were selected, offset by 1,

and log2 transformed. Multiple gene level mappings were resolved by singular value decomposition. Datasets from both tissues were merged, and a custom ComBat correction was performed to account for batch effects between HiSeq-RNASeqV2 and Illumina-GA platforms. All scripting and normalization methods are available for download via the CRC Subtyping Consortium's github including the merging protocol (https://github.com/Sage-Bionetworks/crcsc/blob/dc58542555e281c1ccb55aeb73d087e7d0bdf6bf/groups/G/dataQc/tcgaCrcRNAseq-merged.R) and miscellaneous normalization procedures (https://github.com/Sage-Bionetworks/crcsc/blob/dc58542555e281c1ccb55aeb73d087e7d0bdf6bf/groups/G/dataQc/JGnorm.R).

For both microarray and RNA Sequencing expression data, features were mapped to corresponding Entrez gene IDs using annotation sets provided by Ensembl GRCH38 and Bioconductor including hgu133a.db (version 3.2.3), huex10sttranscriptcluster.db (version 8.6.0), lumiHumanIDMapping (version 1.10.1), or org.Hs.eg.db (version 3.3.0). For multiple annotations mapping to a unique gene feature, either the median probeset value or the largest coefficient of variation across RNAseq samples was retained as an expression estimate for the corresponding gene feature. CMS classification was performed using the single sample procedure (SPP) (https://github.com/Sage-Bionetworks/CMSclassifier). The single sample method is a Pearson correlation-based centroid model of 786 genes which is included with the CMSClassifier R package.

7. Similarity Network Fusion

The matched normalized mRNA and miRNA expression data of 93 metastases were first separately standardized using the standardNormalization function from the R package SNFtool (version 2.2). The Euclidean distances between all pairs of samples in mRNA and miRNA data were calculated, respectively. An affinity matrix was computed using the function affinityMatrix with the number of nearest neighbors K and the variance for local model alpha. We then performed similarity network fusion on affinity matrices of mRNA and miRNA with the number of iterations T, which was used in the subsequent spectral clustering step where samples were assigned to one of the SNF clusters. We identified three clusters using default settings. In order to find other possible compositions of three clusters we tested 168

parameter combinations of K (10, 15, 20, 25, 30, 35, 40), alpha (0.3, 0.4, 0.5, 0.6, 0.7, 0.8), and T (20, 30, 40, 50). For each parameter setting, we applied the estimateNumberOfClustersGivenGraph function to estimate the possible number of clusters using two heuristic methods: (1) eigen gap and (2) rotation cost. We retained the clustering results which comprised three clusters and calculated the median Silhouette index (SI) of each result. We selected the top 8 clustering results that had the highest median SIs (**Supplementary Fig. 5A**). A majority voting scheme was applied to determine the final cluster membership based on the top 8 clustering results. In the event of a tie, we chose the membership defined by four clustering results with the largest median SIs. The association between SNF clusters and clusters derived from mRNA or miRNA consensus clustering were evaluated using Chi-squared tests (**Supplementary Fig. 5B**).

Robustness of SNF Clustering on Overall Survival

Previous work has shown that the SNF algorithm for clustering is statistically robust[11]. We examined whether our observed survival difference between SNF clusters could be reproduced by random chance. To this end, we performed permutation analyses. For each permutation, miRNA profiles were shuffled and randomly assigned to mRNA profiles. Subsequently, SNF clustering was performed *de novo* and each patient was assigned to one of three resulting groups. Differential overall survival across clusters was then assessed with a log-rank test. This process was repeated 1,000 times, and log-rank p-values were used to construct a null distribution. We examined the number of instances when p-values from the null distribution were more extreme (i.e. smaller) than our empirical p-value (**Supplementary Fig. 7**).

8. Ensemble of Gene Set Enrichment Analyses (EGSEA)

Raw gene feature counts were mapped to Entrez ID using the R/Bioconductor package org.Hs.eg.db v3.4.0[5]. Low/non-expressed genes with less than 1 CPM across the minimum number of samples in any subtype group were excluded from subsequent analysis using edgeR v3.16.5. Quality weighted, quantile, and log-normalized CPM were calculated using limma-voom v3.30.11. Gene set enrichment was performed using the R/Bioconductor package EGSEA v1.2.0[12] with planned contrasts of each subtype against the average of the remaining subtypes. Independent EGSEA analyses were performed for gene lists provided by MSigDB v5.2[13] (**Supplementary Table 4**) and a custom gene list identifying numerous

immunological, canonical, and metabolic pathways[14] (**Supplementary Table 5**). Intratumoral immunome profiling was performed as previously described[15], and resulting gene lists were used to calculate subtype-level and single sample enrichment scores using EGSEA.

9. SNF Class Predictor

Data Preprocessing

To build a classifier to distinguish samples between subtype cluster 2 (C2) and subtype clusters 1 and 3 combined (C13), the normalized mRNA expression data of 93 patients was split into a training set, consisting of 20 subtype 2 samples and 51 subtypes 1 and 3 samples, and a test set, consisting of 6 cluster 2 samples and 16 cluster 1 and 3 samples. The class ratio remained unchanged during the partition. For the training set, we first filtered genes with near zero-variance. We then identified highly correlated genes with a pair-wise absolute correlation coefficient greater than 0.7, and removed those with the largest mean absolute correlation. We further removed potential linear dependencies of the data using the findLinearCombos function from the R package Caret (version 6.0). We applied the preProcess function to center and scale the training and test data by mean and standard deviation, followed by rescaling data to -1 and 1.

Model Training and Testing

We applied Prediction Analysis of Microarrays (PAMR, version 1.55) – a nearest shrunken centroid classification algorithm – on the training set[16]. A 10-fold cross-validation was performed to obtain the optimal threshold of 2.72 for the prediction, where the overall error rate was 0.056. The final classification model contains 113 genes and was evaluated using the held-out test data of 6 subtype 2 samples and 16 subtypes 1 and 3 samples. Performance metrics such as accuracy, balanced accuracy, sensitivity, specificity, positive prediction value (PPV), negative prediction value (NPV), Cohen's Kappa, Matthew's correlation coefficient, and area under the curve (AUC) were calculated using the confusionMatrix function from the Caret package and an in-house script (**Supplementary Fig. 11A** and **Supplementary Fig. 11B**).

Independent Validation of Classifier

We downloaded the raw expression data of 96 patients from ArrayExpress (study ID: E-MTAB-1951). We

prioritized the analysis of the E-MTAB-1951 samples as it is the only publicly available colorectal cancer liver metastasis dataset with available clinical annotations (i.e. Clinical Risk Scores (CRS)) to test for association with subtype membership grouping. The samples were profiled using the Illumina HumanHT-12 v3.0 Expression BeadChip. Using the R/Bioconductor package lumi (version 2.26.4)[17], we transformed the expression data via variance-stabilizing transformation (VST) algorithm, followed by between-chip normalization with the robust spline normalization (RSN) algorithm. For multiple probes that mapped to the same Ensembl gene ID, we removed those with the smallest variance across samples. We re-trained the PAM classifier on all 93 samples in our cohort using the 113 genes selected from the previous analysis and applied it to the normalized E-MTAB-1951 microarray data set. For genes that were missing in the microarray data, we replaced the expression values with -1 after scaling the data to -1 and 1. The concordance between the predicted subtype cluster memberships and the Clinical Risk Scores (CRS) from the E-MTAB-1951 samples was examined using contingency analysis (**Supplementary Fig. 11C)**.

10. Hybrid Capture Next Generation Sequencing

<u>Targeted Capture Sequencing Panel</u>

For each specimen, DNA from 1,212 exonic regions was captured using the UCM-OncoPlus panel based on the NimbleGen SeqCap EZ custom capture method as previously described[18]. In brief, this approach utilizes a tiered assay system in which highly clinically relevant genes (tier 1, n=316) are sequenced approximately 3-fold deeper than the remaining (tier 2) genes. Capture libraries were generated using the Illumina TruSeq platform. Libraries were multiplexed with 6 base-paired indexes up to 9 samples per lane and sequenced using Illumina HiSeq2000 and HiSeq2500 machines. FastQ files were generated using Illumina's BCL2FastQ1.8.4.

<u>Sequencing Data Alignment</u>

FastQ files were quality trimmed using cutadapt v1.9.1 (http://cutadapt.readthedocs.io/en/stable/guide.html) for Phred score quality on 3' end Q >=30[18] and a minimum length of 19 after trimming (bwa-mem recommended minimum read size). Remaining reads were aligned using the bwa-mem algorithm v0.7.8 (http://bio-bwa.sourceforge.net) against the hg19 reference. PCR duplicates were removed by Broad Institute Picard tools v1.128 MarkDuplicates

(https://github.com/broadinstitute/picard). Bedtools v2.22.1 (http://bedtools.readthedocs.io/en/latest/) was used to ascertain coverage at tier 1 and tier 2 loci. Samples that did not have a mean 300X depth of coverage at tier 1 genes were excluded from subsequent analyses. In targeted-capture sequencing, oxidative damage can be pervasive and lead to false positive variant calls at sites with sequence context CCG being read as CAG[19]. Sample-level oxidative damage was calculated using Picard CollectOxoGMetrics. Sample with ArtQ[19] scores less than 21 were removed. Overall, 59 unique metastasis-normal pairs were available for analysis.

Example alignment pipeline flow:

1. cutadapt -m 19 --quality-base=33 -q30 -a ZZZ-A ZZZ -o sample_filtered_r1.fastq.gz -p sample_filtered_r2.fastq.gz sample_r1.fastq.gz sample_r2.fastq.gz >> sample.cutadapt.log 2>> sample.cutadapt.log

2. bwa mem -t 8 -R "@RG\tID:sample_flowcell_id\tLB:sample\tSM:sample\tPL:illumina" -v 2 hg19.fa sample_filtered_r1.fastq.gz sample_filtered_r2.fastq.gz | samtools view -bT hg19.fa - > sample.bam) > sample.bwa.pe.log 2>&1

3. novosort -c 8 -m 30G --tmpdir novosort_tmp -o sample.srt.bam -i sample.bam > sample.novosort.log 2>&1

4. java -Xmx30g -jar picard.jar MarkDuplicates CREATE_INDEX=true TMP_DIR=picard_tmp REMOVE_DUPLICATES=true ASSUME_SORTED=true MAX_FILE_HANDLES_FOR_READ_ENDS_MAP=500 INPUT=sample.srt.bam OUTPUT=sample.rmdup.srt.bam METRICS_FILE=sample.rmdup.srt.metrics VALIDATION_STRINGENCY=LENIENT > sample.picard.rmdup.pe.log 2>&1

5. bedtools coverage -hist -abam sample.rmdup.srt.bam -b capture_panel_v3_t1.bed | grep all > sample.capture_t1.hist

6. bedtools coverage -hist -abam sample.rmdup.srt.bam -b capture_panel_v3_t2.bed | grep all > sample.capture_t2.hist

7. java -Xmx30g -jar picard.jar CollectOxoGMetrics I=sample.rmdup.srt.bam O=sample.oxo_summary.txt R=hg19.fa INTERVALS=capture_panel_v3.intvl 2> sample.oxo.log;

Variant Calling and Filtering

Single nucleotide variants (SNVs) were called using MuTect v1.1.7 (http://archive.broadinstitute.org/cancer/cga/mutect). Insertions and deletions (indels) were called using scalpel-discovery 0.5.3 (http://scalpel.sourceforge.net/). Calls not annotated as “PASS” or “KEPT” were removed. For both SNVs and indels, only calls falling within genomic coordinates targeted by the capture panel were retained for subsequent analyses. Targeted capture libraries have been shown to be susceptible to oxidative damage. Even samples that do not have pervasive oxidative damage can have

false positive calls attributable to this phenomenon[19]. All SNV calls were assigned a FoxoG score using metalfox (https://github.com/cpwardell/bin/blob/master/metalfox.py). Based on previously reported studies[19], calls without a MuTect tumor_lod greater than -10 + (100 / 3) * FoxoG were removed as they were likely a consequence of oxidative damage. All variants were annotated using snpeff v3.6c (http://snpeff.sourceforge.net/), hg19 reference. Only variants that exist within coding regions or disrupted splice sites were included in analyses. Calls with a variant allele frequency (VAF) < 5%, position coverage < 30, or an allele frequency >= 0.01 in ExAC were removed. To further improve the quality of indel calls, two additional filters were implemented: (1) Dustmasker (https://www.ncbi.nlm.nih.gov/IEB/ToolBox/CPP_DOC/lxr/source/src/app/dustmasker/) was used to identify low complexity genomic regions, and indels falling within these regions were discarded; (2) A pseudo-panel of normal samples was constructed, such that across the matched normal samples, all putative indel calls that failed Scalpel filters due to 'HighVafNormal' or 'HighAltCountNormal' were aggregated. All indels that failed in two or more samples from unique patients were filtered. These methods helped to eliminate remaining noisy calls which passed previous filtering steps.

Example variant calling workflow:

1. java -Djava.io.tmpdir=./temp -Xmx2g -jar mutect-1.1.7.jar -T MuTect -R hg19 --intervals chr1.intvl --input_file:normal normal_bam tumor_bam --max_alt_alleles_in_normal_count 1000 --max_alt_alleles_in_normal_qscore_sum 37 --max_alt_allele_in_normal_fraction 0.05 --out tumor_normal/tumor_normal.chr1.out -vcf tumor_normal/tumor_normal.chr1.vcf --enable_extended_output --strand_artifact_power_threshold 0 -log tumor_normal.mutect.chr1.log 2>> 'tumor_normal.mutect.chr1.log;

2. metalfox.py -f1 tumor_normal/tumor_normal.chr1.out.keep -f3 tumor.bam -m wgEncodeCrgMapabilityAlign100mer.bedGraph.gz > tumor_normal.foxog_scored_added.out

3. scalpel-discovery --somatic --logs --numprocs 8 --tumor tumor_bam --normal normal_bam --bed capture_panel_v3.bed --ref hg19.fa 2>> tumor_normal.indels.log

4. java -jar snpEff.jar eff -t hg19 (tumor_normal.out.keep.sift.vcf/tumor_normal.somatic_indel.PASS.sift.vcf) -v > (tumor_normal.out.keep.eff.vcf/tumor_normal.somatic_indel.PASS.eff.vcf) 2>> tumor_normal.snpeff.log

Mutation Significance (MutSig) Analysis

VCFs were annotated and converted to a MAF format using Oncotator[20]. MAF files for all patients were merged and assessed for significant gene-centric mutation frequency using MutSigCV version 2 with

default coverage and covariate tables provided by the Broad Institute[21]. Mutation Assessor[22] and ClinVar[23] were used to predict the functional impact of protein-coding mutations.

Copy Number Variation Analysis

Copy number calling was carried out using CNVKit v0.7.12.dev0[24]. All 59 matched-normal samples were used to calculate the pooled reference baseline using default parameters. Segmented log2 ratios were used to call copy number gains and losses.

Identification of Prognostic Mutations

Multivariate Cox proportional hazard ratios were generated for each mutated gene feature as a binary factor across 59 liver metastasis-matched paired normal samples using the survival v2.40-1 R package. Molecular subtype and Clinical Risk Score (CRS) were included as covariates in multivariate analyses. Ten-year overall survival was chosen as the primary endpoint of the analysis.

11. Microsatellite Instability (MSI) Analysis

H&E slides of normal and tumor specimens were reviewed by a molecular pathologist (Dr. Nora Joseph). Tumor sections with greater than 30% tumor percentage were used for DNA extraction by the Pinpoint Slide DNA Isolation System (Zymo Research). DNA was subsequently purified by using the Zymo-Spin I Column protocol. All samples were run on the Promega MSI 1.2 assay according to the FDA approved protocol and result interpretation. MSI testing was performed on 93 metastases with corresponding molecular subtypes of which 89 samples were successfully assayed. Four samples failed repeated testing.

12. Immunohistochemical Analysis

CRC liver metastases were preserved in formalin and embedded in paraffin. 5μm tissue sections were created from paraffin blocks and mounted on glass slides. The slides were stained on Leica Bond RX Automatic Stainer using HTRC Bond Refine DAB protocol. After antigen retrieval treatment (epitope retrieval solution II, AR9640, Leica Biosystems) for 20 minutes, anti-human CD3 (DAKO, Cat#M7254, Clone: F7.2.38, mouse IgG) antibody (1:600) was applied on tissue sections for 25 minutes incubation. For CD8 staining, anti-human CD8 (DAKO, Cat#M7103, Clone: C8/144B, mouse IgG) antibody (1:400)

was applied. The antigen-antibody binding was detected with Bond polymer refine detection (Leica Biosystems, DS9800). A coverslip was applied to the tissue sections. For Masson's trichrome staining, tissue sections were deparaffinized using heated Bouin's solution and then stained with Weigert's iron hematoxylin and Biebrich scarlet solutions. The tissue sections were then treated with phosphotungstic-phosphomolybdic acid and immediately stained with aniline blue solution. The tissue sections were rinsed and a coverslip was applied.

B. Supplementary Figures

Supplementary Figure 1: Overview of study design.

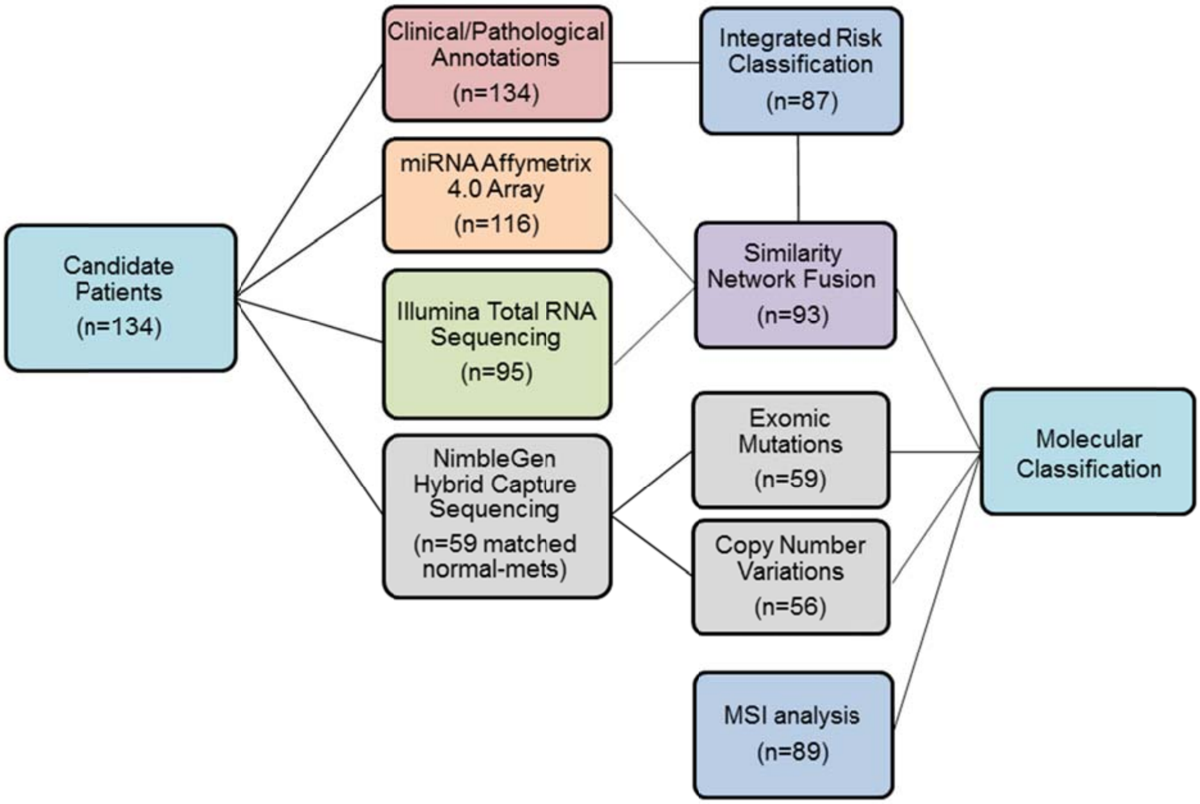

Supplementary Figure 2: Overall survival by Consensus Molecular Subtypes (CMS) in patients with colorectal liver metastases. CMS subtypes were determined for 93 patients in our cohort from RNA Sequencing data using the methodology implemented in Sage-Bionetwork's CMSclassifier R package (see **Supplementary Methods**). Kaplan-Meier survival analysis of 10-year overall survival was performed for patients with CMS2, CMS4 and unclassified patterns. One patient with a CMS1 pattern was excluded from survival analysis. No. at risk denotes the number of patients at risk at each specified time point. P-value was determined using a log-rank test across groups.

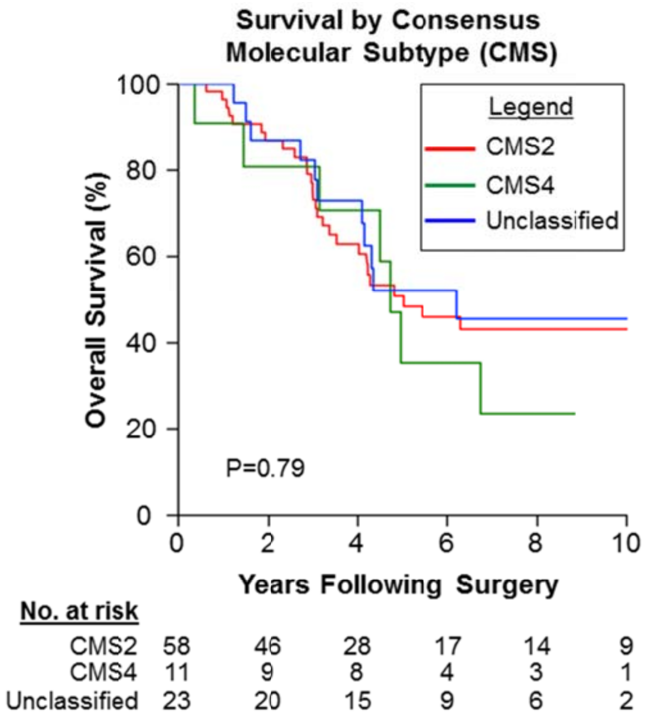

Supplementary Figure 3: Consensus clustering analysis of the mRNA expression data for 95 patients with colorectal liver metastases. (A) Heatmaps of the consensus matrices for the predefined cluster numbers k (k = 2, 3, 3, 4, 5, and 6); **(B)** Kaplan-Meier plot for 10-year overall survival of the patients stratified by their consensus cluster memberships. P-value was determined using a log-rank test across groups; **(C)** Consensus Cumulative Distribution Function (CDF) plot of the consensus matrix for each k, estimated by a histogram of 100 bins. The lower left portion of the CDF plot represents samples rarely clustered together, and the upper right portion represents those almost always clustered together, whereas the middle portion represents those with occasional co-assignments in different clustering runs; A flat middle segment, suggesting that very few sample pairs are ambiguous when k is correctly inferred, can be used to determine the optimal k of consensus clusters. **(D)** Proportion of ambiguous clustering (PAC) plot defined as the fraction of sample pairs with consensus index values falling in the intermediate sub-interval (0.1, 0.9). A low value of PAC indicates a flat middle segment in the CDF plot and is allowed to infer the optimal k (k = 2).

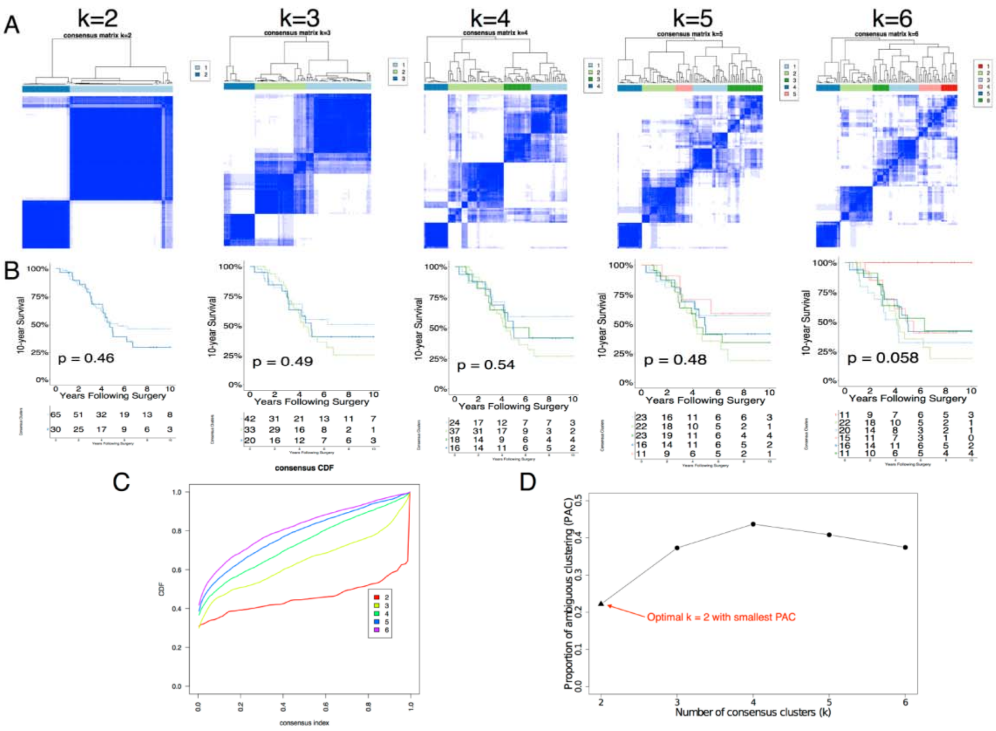

Supplementary Figure 4: Consensus clustering analysis of the miRNA expression data for 116 patients with colorectal liver metastases. (A) Heatmaps of the consensus matrices for the predefined cluster numbers k (k = 2, 3, 3, 4, 5, and 6); **(B)** Kaplan-Meier plot for 10-year overall survival of the patients stratified by their consensus cluster memberships. P-value was determined using a log-rank test across groups; **(C)** Consensus Cumulative Distribution Function (CDF) plot of the consensus matrix for each k, estimated by a histogram of 100 bins. The lower left portion of the CDF plot represents samples rarely clustered together, and the upper right portion represents those almost always clustered together, whereas the middle portion represents those with occasional co-assignments in different clustering runs; A flat middle segment suggesting that very few sample pairs are ambiguous when k is correctly inferred, can be used to determine the optimal k of consensus clusters. **(D)** Proportion of ambiguous clustering (PAC) plot defined as the fraction of sample pairs with consensus index values falling in the intermediate sub-interval (0.1, 0.9). A low value of PAC indicates a flat middle segment in the CDF plot and is allowed to infer the optimal k (k = 2).

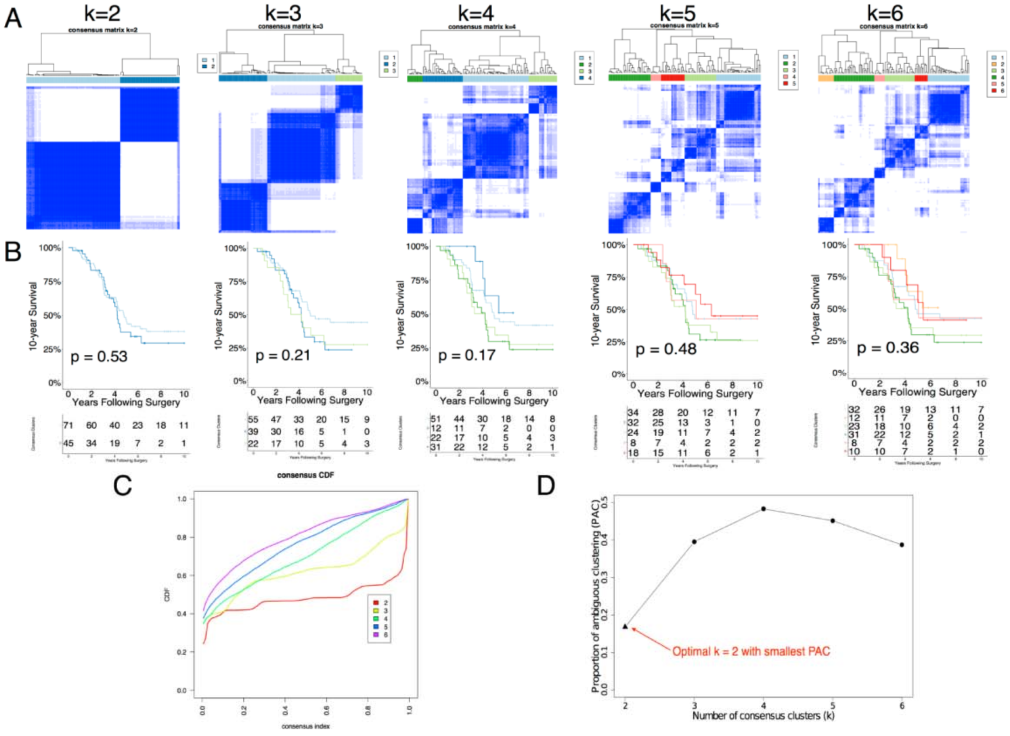

Supplementary Figure 5: Median Silhouette Index (SI) for the SNF clusters under 72 parameter settings. (A) SI represents the separation distance between the resulting clusters under each parameter setting. The top 8 parameter settings with highest median SI (in red) were selected for further analysis, and the corresponding clustering results were used to determine the final SNF cluster memberships through majority voting. Statistical differences in the associations between SNF clusters (S1=Subtype 1; S2= Subtype 2; S3= Subtype 3) and mRNA **(B)** or miRNA **(C)** consensus clusters (C1-C4) for pre-defined consensus cluster numbers of k=2, k=3, and k=4 were assessed using Chi-squared tests across SNF clusters. Asterisks denote P-values ≤ 0.0001.

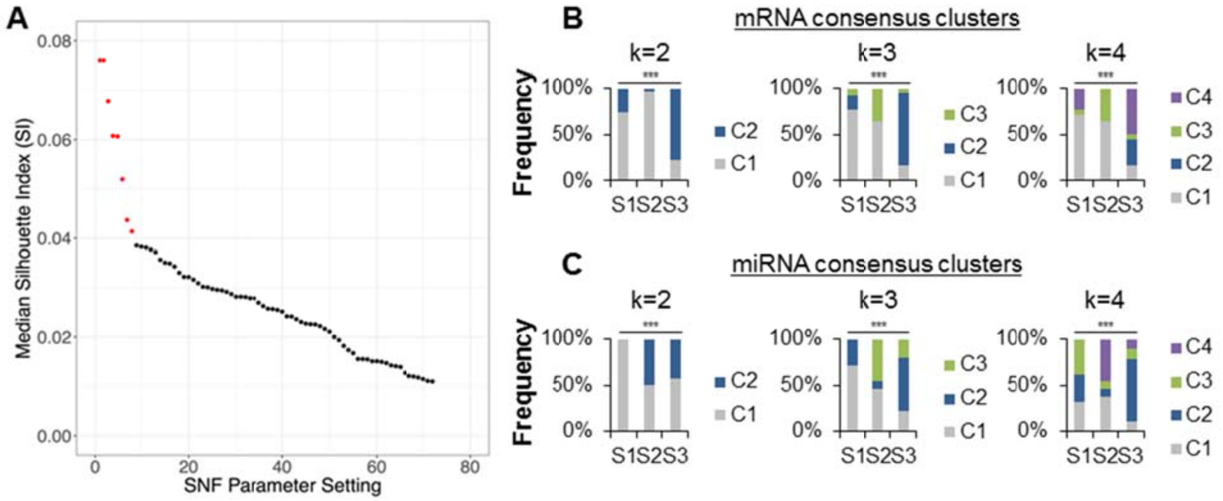

Supplementary Figure 6: Associations of molecular subtypes and clinicopathological variables. Statistical significance was assessed using Fisher's exact tests for categorical variables and Student's t-test for continuous variables. Asterisks denote P-values ≤ 0.05 in the comparison of one SNF subtype versus the remaining subtypes. Error bars represent S.E.M..

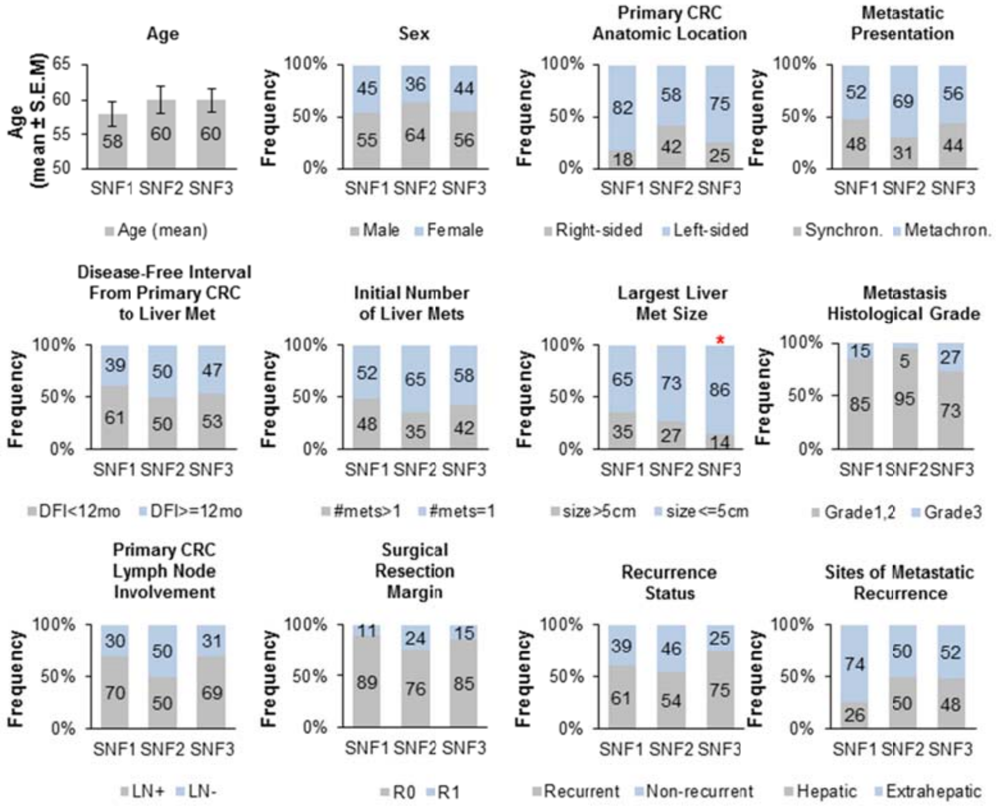

Supplementary Figure 7: Non-random association of SNF network structure with overall survival in metastatic colorectal cancer patients. Shown is the density of the -log P-value for each simulation of the SNF cluster set with members closest to the consensus for illustrative purposes. The red line in the figure represents the empirical P-value for a particular parameter set (parameter settings: K=25, alpha=0.6, T=20). The table inset contains the key statistics for each of the top 8 SNF cluster parameterizations in order of decreasing median Silhouette Index; highlighted is a parameter setting which produced SNF clusters with memberships closest to the consensus SNF grouping differing only by two sample assignments.

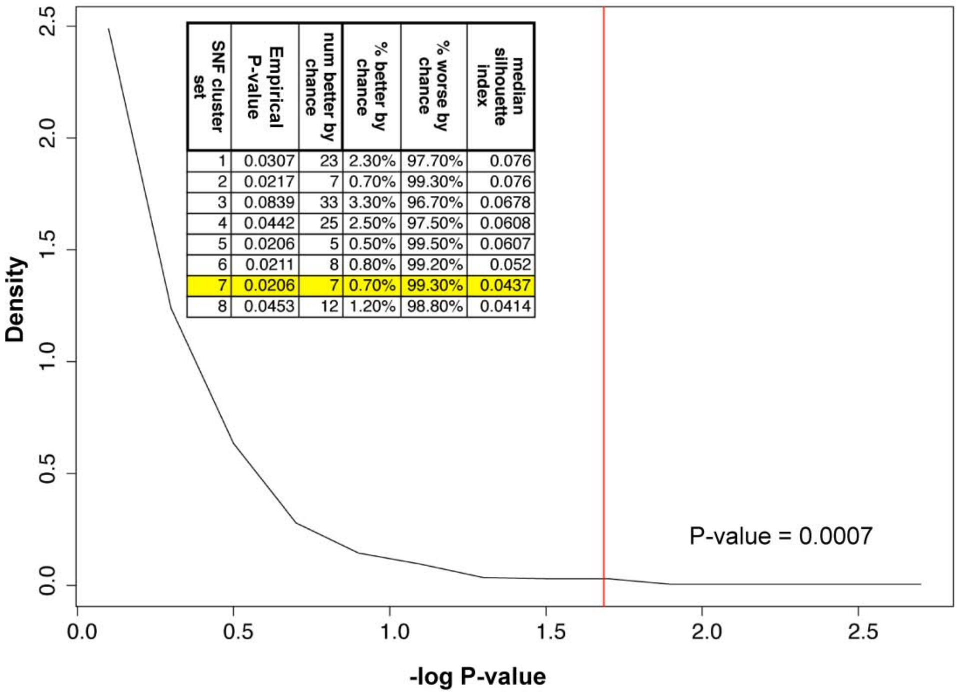

SNF cluster set	Empirical P-value	num better by chance	% better by chance	% worse by chance	median silhouette index
1	0.0307	23	2.30%	97.70%	0.076
2	0.0217	7	0.70%	99.30%	0.076
3	0.0839	33	3.30%	96.70%	0.0678
4	0.0442	25	2.50%	97.50%	0.0608
5	0.0206	5	0.50%	99.50%	0.0607
6	0.0211	8	0.80%	99.20%	0.052
7	0.0206	7	0.70%	99.30%	0.0437
8	0.0453	12	1.20%	98.80%	0.0414

Supplementary Figure 8: Distant metastasis-free survival by molecular subtype. Molecular subtypes were determined for 93 patients in our cohort. Subtype 1 = SNF1, Subtype 2 = SNF2, and Subtype 3 = SNF3. Kaplan-Meier survival analysis of distant metastasis-free survival (event = first metastatic recurrence or death). No. at risk denotes number of patients at risk at each specified time point. P-value was determined using a log-rank test across groups.

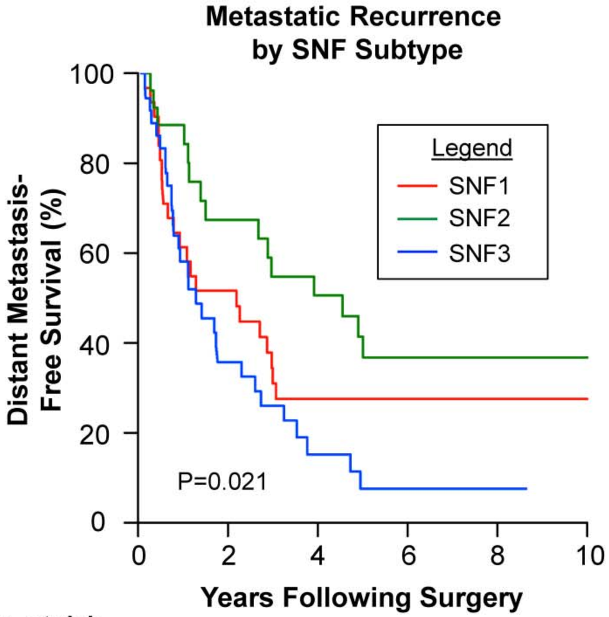

No. at risk

SNF1	31	16	9	7	5	2
SNF2	26	17	13	9	9	5
SNF3	36	12	5	3	2	1

Supplementary Figure 9: Primary CRC CMS subtype by metastasis subtype. Shown is the distribution of primary colorectal cancer Consensus Molecular Subtypes (CMS) by SNF-based molecular subtypes of colorectal liver metastases. Subtype 1 = SNF1, Subtype 2 = SNF2, and Subtype 3 = SNF3. CMS subtypes were determined for 93 patients in our cohort from RNA Sequencing data using the methodology implemented in Sage-Bionetwork's CMSclassifier R package (see **Supplementary Methods**). P-value denotes a Chi-Squared test across the three SNF groups.

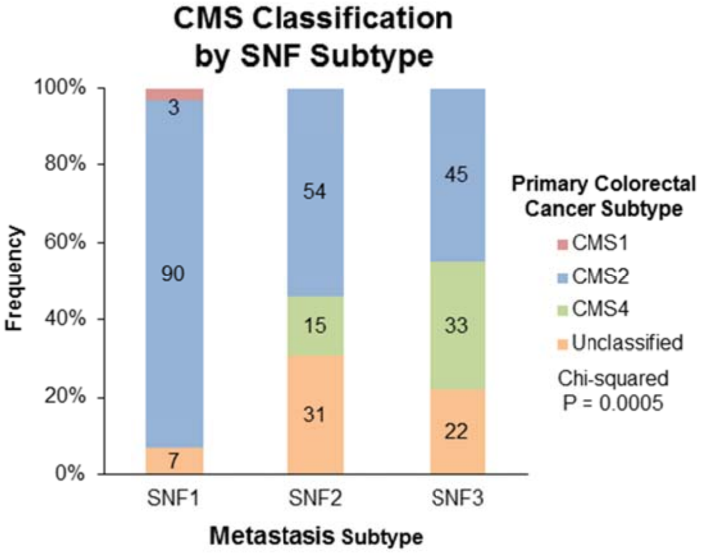

Supplementary Figure 10: Perioperative chemotherapy regimens and associations with molecular subtype. (A) Types of perioperative chemotherapies received by patients which were included in the integrated SNF-based molecular analysis. Specific details regarding chemotherapy regimens were available for 81 of 93 patients. **(B)** Association between type of chemotherapy received in perioperative setting and molecular subtype of metastasis derived from molecular subtyping analysis. P-value denotes a Chi-Squared test across the three SNF groups. Subtype 1 = SNF1, Subtype 2 = SNF2, and Subtype 3 = SNF3.

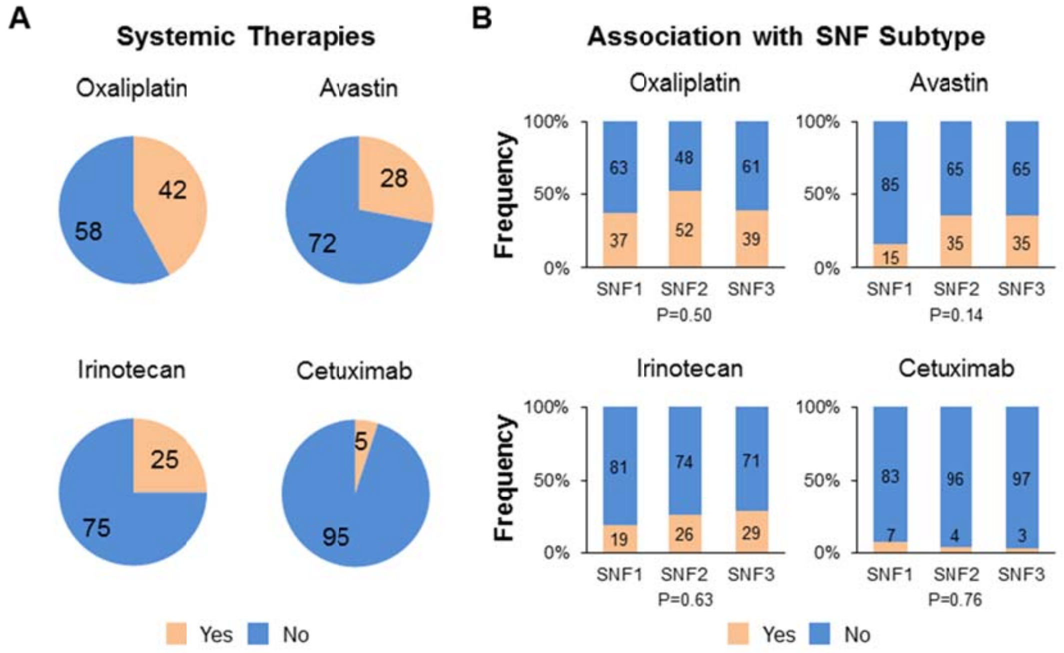

Supplementary Figure 11: Prediction Analysis of Microarrays (PAM)-based classifier to distinguish molecular subtypes. (A) Model evaluation on the test data set from our cohort samples. **(B)** The area under the ROC curve demonstrates the classification performance compared to a random classifier on the test data set (AUC = 0.875 vs. AUC = 0.50 for random classifier). **(C)** Mosaic plot showing the concordance between the predicted subtype cluster labels and the Clinical Risk Scores (CRS) in an independent data set of patients who underwent hepatic resection of limited colorectal liver metastases (Memorial Sloan-Kettering Cancer Center, n=96, ArrayExpress Identifier: E-MTAB-1951). Subtype 1 = SNF1, Subtype 2 = SNF2, and Subtype 3 = SNF3.

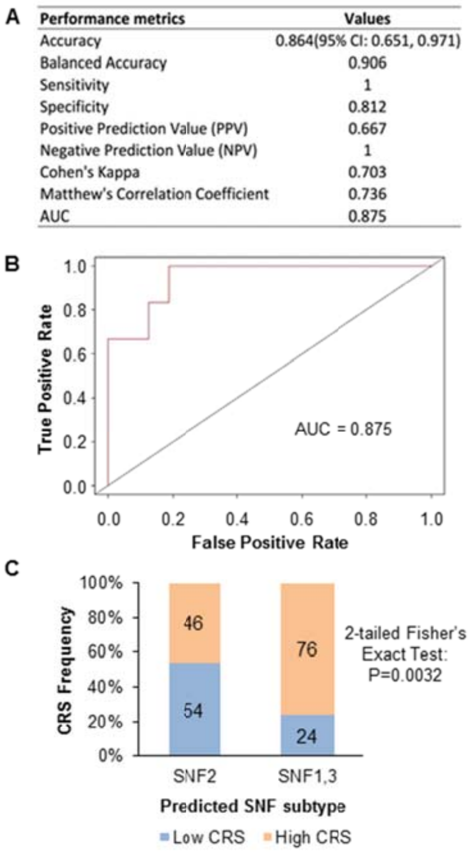

Supplementary Figure 12: Histologic analysis by molecular subtypes of liver metastasis. (A) Hematoxylin and eosin, **(B)** Trichome, **(C)** CD3, and **(D)** CD8 staining by subtype. Shown are 10X magnification fields for three representative patients from each SNF subtype. Top row, Subtype 1. Middle row, Subtype 2. Bottom row, Subtype 3.

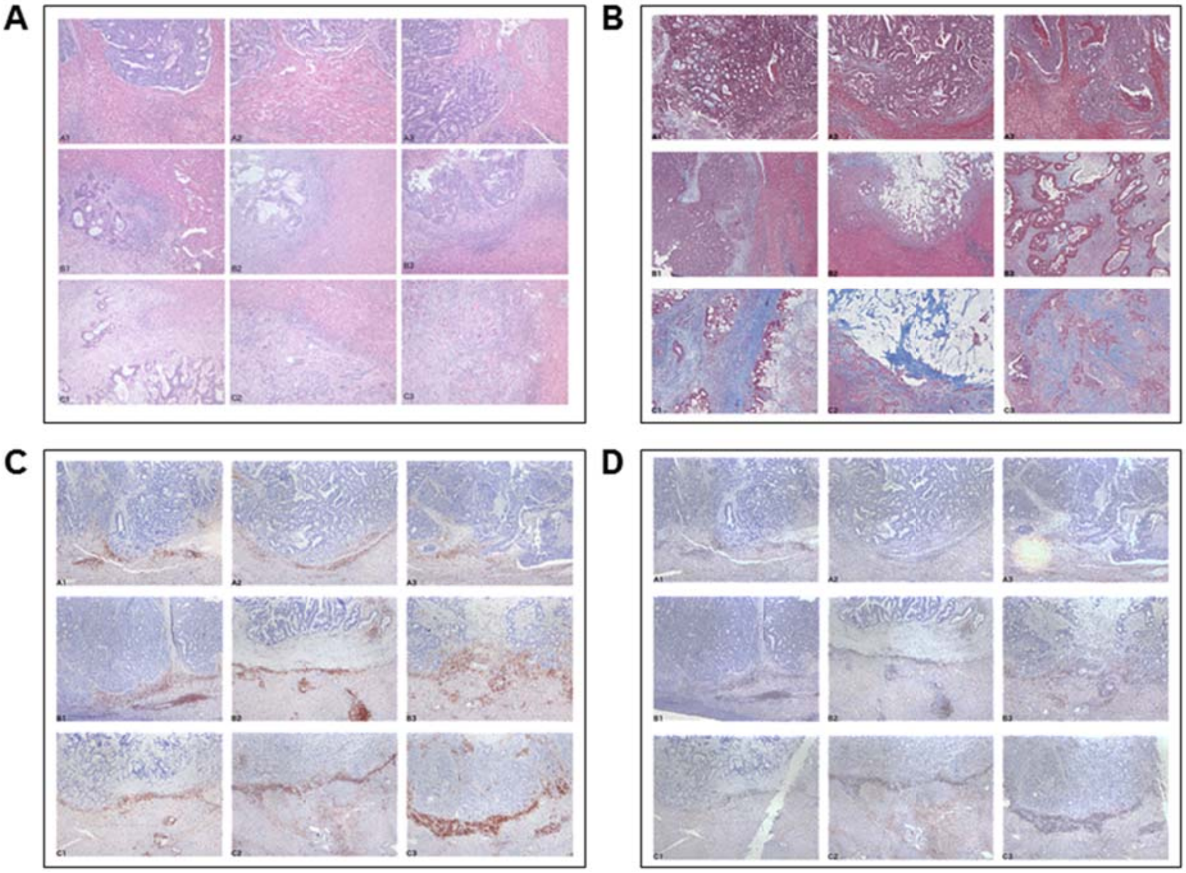

Supplementary Figure 13: Oncoprint plot of exomic mutations occurring in 59 patients with colorectal liver metastases. Genes mutated in ≥ 10% of samples are shown. Values to the left of the Oncoprint plot represent the percentage of samples that harbor a mutation (non-synonymous SNVs or indels) in a given gene. The horizontal bar plot indicates the number of mutations for each patient sample falling within these recurrently altered genes. The vertical bar plot to the right depicts the number of mutations seen in each gene across all 59 samples. 'Splicing' refers to mutations that affect a splice donor or acceptor site.

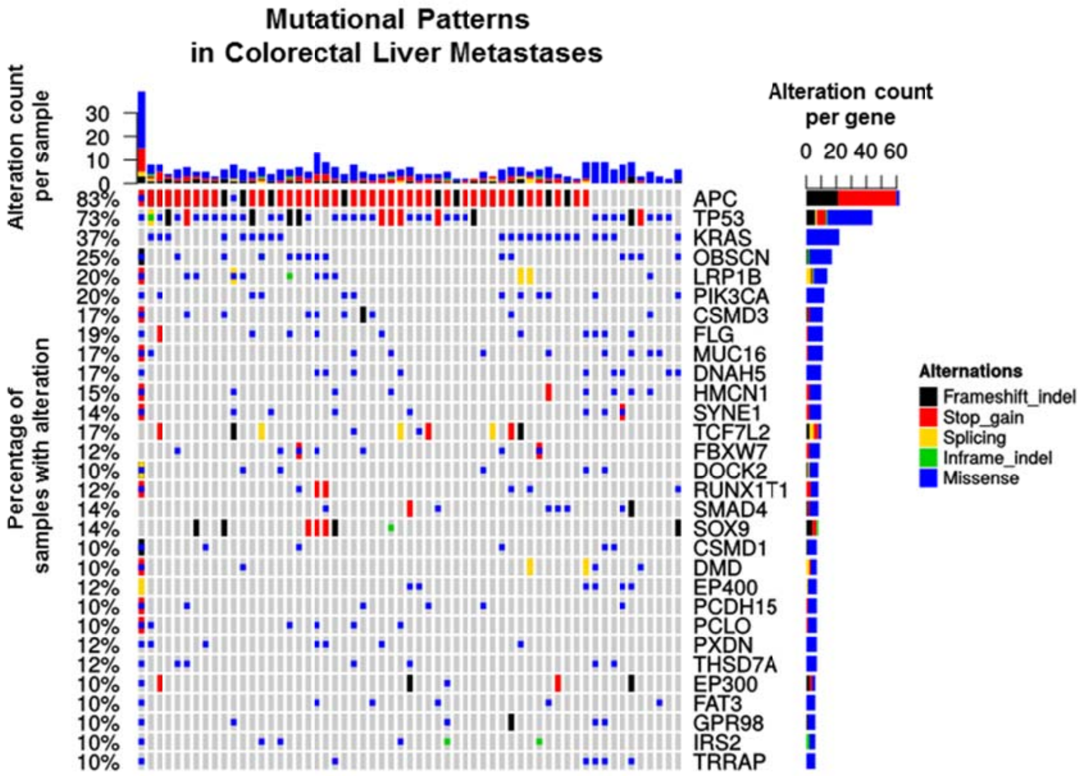

Supplementary Figure 14: Cytotoxic immune signature by molecular subtypes. (A) Distribution of cytotoxic immune gene scores[15] by CRC liver metastasis subtype. MSI-H and MSI-L, microsatellite instability-high and -low. MSS, microsatellite stable. N/A, missing data. Subtype 1 = SNF1, Subtype 2 = SNF2, and Subtype 3 = SNF3. **(B)** Mean (± S.E.M.) values of cytotoxic cell immune scores by SNF-based molecular subtype. **(C)** Percentage of MSS patients within each SNF-derived subtype. Differences in cytotoxic immune scores by somatic *ARID2* **(D)** or SNF-specific mutations **(E)**. Metastases classified as harboring SNF subtype 2-specific mutations included *CDK12*, *NRAS*, and *EBF1* mutations, whereas *SMAD3*, *NOTCH1*, or *PIK3C2B* mutations characterized SNF subtypes 1- and 3-specific mutations. Data represent mean ± S.E.M. values. Asterisks denote P-values ≤ 0.05 based on two-tailed Student's t-test.

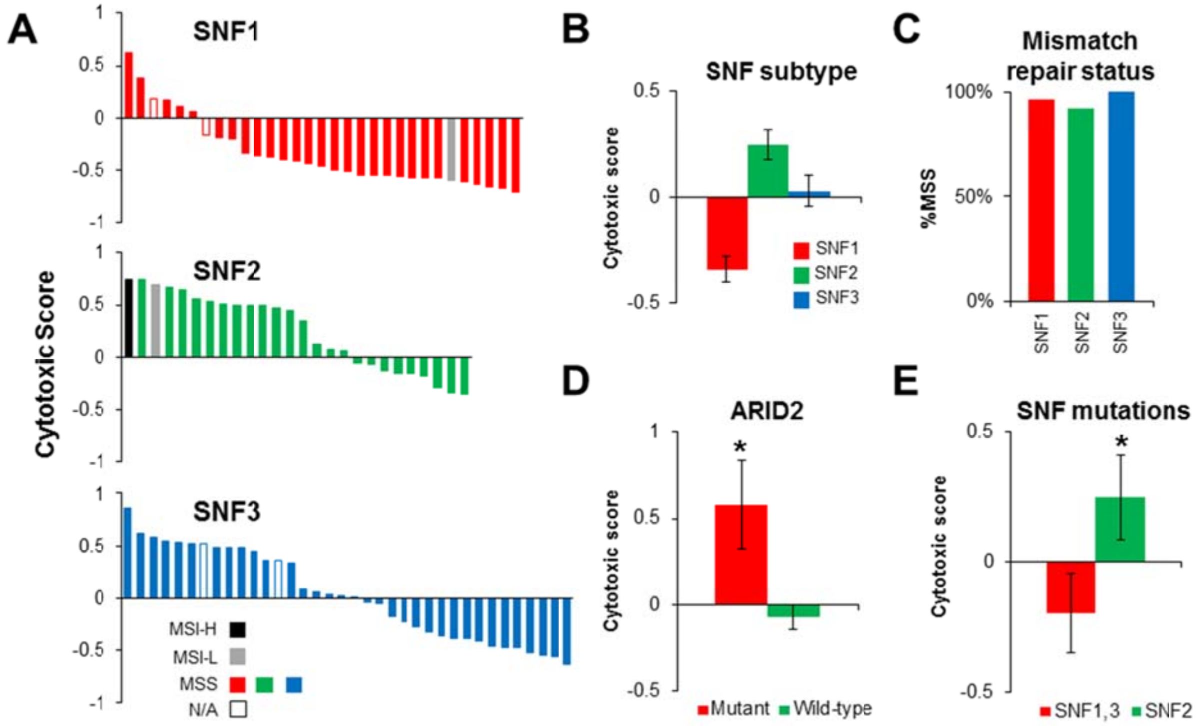

Supplementary Figure 15: Overall survival by integration of molecular subtype and Clinical Risk Scores (CRS). High CRS denotes scores ≥ 2. Patient subgroups defined by molecular subtypes and CRS were classified into low-, intermediate-, and high-risk cohorts based on Kaplan-Meier analysis of overall survival rates. P-value was determined using a log-rank test across groups. No. at risk denotes number of patients at risk at each specified time point. Table inset denotes hazard ratios (95% confidence intervals) for Cox multivariate proportional hazard analysis of molecular subtype (3 levels; SNF1 vs. SNF2 vs. SNF3) and CRS (2 levels; CRS >=2 vs. CRS <2). Both subtype and CRS were considered nominal variables. A multivariate interaction was assessed between subtype and CRS and removed from the final multivariate model due to non-significance. P-value was determined using likelihood ratio test. The differences in CRS between SNF subtypes were assessed using Chi-Squared tests. Asterisk denotes P-values ≤ 0.05 in the comparison of one subtype versus the remaining subtypes.

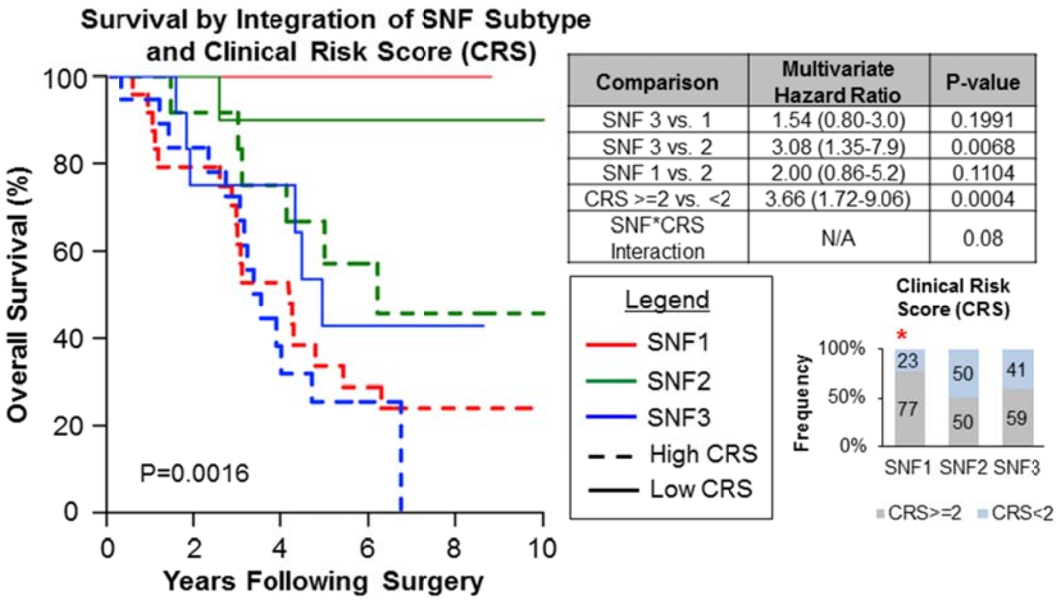

Comparison	Multivariate Hazard Ratio	P-value
SNF 3 vs. 1	1.54 (0.80-3.0)	0.1991
SNF 3 vs. 2	3.08 (1.35-7.9)	0.0068
SNF 1 vs. 2	2.00 (0.86-5.2)	0.1104
CRS >=2 vs. <2	3.66 (1.72-9.06)	0.0004
SNF*CRS Interaction	N/A	0.08

**No. at risk**							
SNF1-lowCRS	7	7	5	4	2	1	**Low-Risk**
SNF2-lowCRS	12	11	8	8	8	4	
SNF2-highCRS	12	12	10	6	5	3	**Intermediate-Risk**
SNF3-lowCRS	13	10	8	5	2	1	
SNF1-highCRS	24	19	13	7	5	3	**High-Risk**
SNF3-highCRS	19	16	7	2	1	1	

Supplementary Figure 16: Metastatic recurrence patterns by integrated risk classification. Risk groups were determined for 87 patients in our cohort. **(A)** Kaplan-Meier survival analysis of distant metastasis-free survival (event = first metastatic recurrence or death). P-value was determined using a log-rank test across groups. No. at risk denotes number of patients at risk at each specified time point. **(B)** Association of molecular/clinical risk stratification groups with patterns of metastatic recurrence. Statistical significance was assessed using Fisher's exact tests between one risk group versus the two remaining groups. Asterisks denote P-values ≤ 0.05.

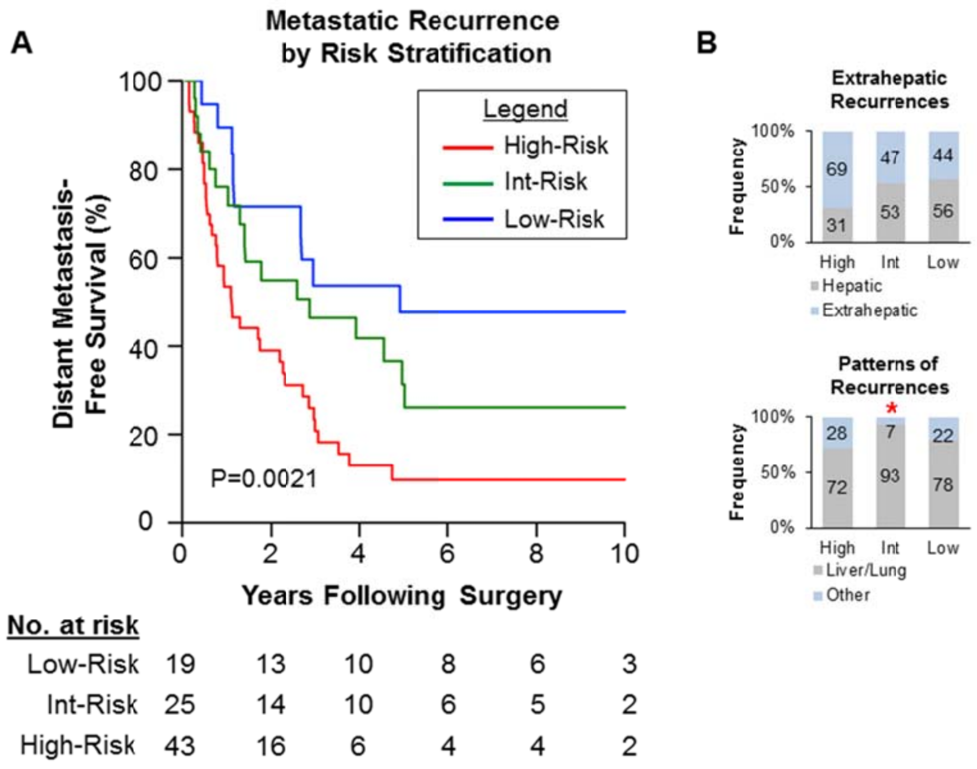

C. Supplementary References

1. Fong Y, Fortner J, Sun RL, Brennan MF, Blumgart LH. Clinical score for predicting recurrence after hepatic resection for metastatic colorectal cancer: analysis of 1001 consecutive cases. Ann Surg 1999;230:309-18; discussion 18-21.

2. Dobin A, Davis CA, Schlesinger F, et al. STAR: ultrafast universal RNA-seq aligner. Bioinformatics 2013;29:15-21.

3. Tarasov A, Vilella AJ, Cuppen E, Nijman IJ, Prins P. Sambamba: fast processing of NGS alignment formats. Bioinformatics 2015;31:2032-4.

4. Liao Y, Smyth GK, Shi W. The Subread aligner: fast, accurate and scalable read mapping by seed-and-vote. Nucleic acids research 2013;41:e108.

5. Robinson MD, McCarthy DJ, Smyth GK. edgeR: a Bioconductor package for differential expression analysis of digital gene expression data. Bioinformatics 2010;26:139-40.

6. Ritchie ME, Phipson B, Wu D, et al. limma powers differential expression analyses for RNA-sequencing and microarray studies. Nucleic acids research 2015;43:e47.

7. Leek JT, Johnson WE, Parker HS, Jaffe AE, Storey JD. The sva package for removing batch effects and other unwanted variation in high-throughput experiments. Bioinformatics 2012;28:882-3.

8. Wu D, Hu Y, Tong S, Williams BR, Smyth GK, Gantier MP. The use of miRNA microarrays for the analysis of cancer samples with global miRNA decrease. Rna 2013;19:876-88.

9. Johnson WE, Li C, Rabinovic A. Adjusting batch effects in microarray expression data using empirical Bayes methods. Biostatistics 2007;8:118-27.

10. Senbabaoglu Y, Michailidis G, Li JZ. Critical limitations of consensus clustering in class discovery. Scientific reports 2014;4:6207.

11. Wang B, Mezlini AM, Demir F, et al. Similarity network fusion for aggregating data types on a genomic scale. Nat Methods 2014;11:333-7.

12. Alhamdoosh M, Ng M, Wilson NJ, et al. Combining multiple tools outperforms individual methods in gene set enrichment analyses. Bioinformatics 2017;33:414-24.

13. Liberzon A, Subramanian A, Pinchback R, Thorvaldsdottir H, Tamayo P, Mesirov JP. Molecular signatures database (MSigDB) 3.0. Bioinformatics 2011;27:1739-40.

14. Guinney J, Dienstmann R, Wang X, et al. The consensus molecular subtypes of colorectal cancer. Nat Med 2015;21:1350-6.

15. Bindea G, Mlecnik B, Tosolini M, et al. Spatiotemporal dynamics of intratumoral immune cells reveal the immune landscape in human cancer. Immunity 2013;39:782-95.

16. Tibshirani R, Hastie T, Narasimhan B, Chu G. Diagnosis of multiple cancer types by shrunken centroids of gene expression. Proceedings of the National Academy of Sciences of the United States of America 2002;99:6567-72.

17. Du P, Kibbe WA, Lin SM. lumi: a pipeline for processing Illumina microarray. Bioinformatics 2008;24:1547-8.

18. Kadri S, Long BC, Mujacic I, et al. Clinical Validation of a Next-Generation Sequencing Genomic Oncology Panel via Cross-Platform Benchmarking against Established Amplicon Sequencing Assays. J Mol Diagn 2017;19:43-56.

19. Costello M, Pugh TJ, Fennell TJ, et al. Discovery and characterization of artifactual mutations in deep coverage targeted capture sequencing data due to oxidative DNA damage during sample preparation. Nucleic acids research 2013;41:e67.

20. Ramos AH, Lichtenstein L, Gupta M, et al. Oncotator: cancer variant annotation tool. Hum Mutat 2015;36:E2423-9.

21. Lawrence MS, Stojanov P, Polak P, et al. Mutational heterogeneity in cancer and the search for new cancer-associated genes. Nature 2013;499:214-8.

22. Reva B, Antipin Y, Sander C. Predicting the functional impact of protein mutations: application to cancer genomics. Nucleic acids research 2011;39:e118.

23. Landrum MJ, Lee JM, Riley GR, et al. ClinVar: public archive of relationships among sequence variation and human phenotype. Nucleic acids research 2014;42:D980-5.

24. Talevich E, Shain AH, Botton T, Bastian BC. CNVkit: Genome-Wide Copy Number Detection and Visualization from Targeted DNA Sequencing. PLoS Comput Biol 2016;12:e1004873.

25. Cancer Genome Atlas N. Comprehensive molecular characterization of human colon and rectal cancer. Nature 2012;487:330-7.
