# Supplementary material for: Integrated molecular subtyping defines a curable oligometastatic state in colorectal liver metastasis
Source: Nat Commun. 2018 May 4;9:1793. doi: 10.1038/s41467-018-04278-6 (PMC5935683; doi:10.1038/s41467-018-04278-6)
Supplement: Supplementary file 3 — Description of Additional Supplementary Files [file 41467_2018_4278_MOESM3_ESM.docx]

**Supplementary Data 1: Samples utilized for genome-wide analyses.**

**Supplementary Data 2. Differentially expressed genes across SNF clusters in 93 metastatic RNA Sequencing samples identified by the limma-voom method.**

**Supplementary Data 3: Differentially expressed miRNAs across SNF clusters in 93 metastatic miRNA samples identified by the limma method.**

**Supplementary Data 4: Ensemble of gene set enrichment analyses for hallmark mSigDB pathway signatures.** Pathway enrichment or depletion (i.e., direction; Dir) was determined for each SNF cluster against the others (e.g., (SNF1 – (SNF2 + SNF3) / 2)). The Hallmark Signature gene list was retrieved from Broad Institute’s mSigDB. Twelve gene set enrichment algorithms (including GSVA, GAGE, PADOG, etc.) were used for analyses, and run independently for each set of gene lists. Raw P-values for a given pathway were combined across algorithms using Fisher’s method and adjusted for multiple testing corrections by Bonferroni’s method. Log2 transformed fold-change (Log2FC) was averaged in a similar fashion. A collective significance score (Sig) proportional to combined P-values and average Log2FC was generated and scaled from 0-100 to assess the degree of pathway enrichment or depletion relative to the inclusive set.

**Supplementary Data 5: Ensemble of gene set enrichment analyses for custom colorectal cancer pathways.** Pathway enrichment or depletion (i.e., direction; Dir) was determined for each SNF against the others (e.g., (SNF1 – (SNF2 + SNF3) / 2)). A compilation of pathways associated immunology, metabolism, canonical pathways, cancer signatures, and stromal infiltration estimates were retrieved from^14^. Twelve gene set enrichment algorithms (including GSVA, GAGE, PADOG, etc.) were used for analyses, and run independently for each set of gene lists. Raw P-values for a given pathway were combined across algorithms using Fisher’s method and adjusted for multiple testing corrections by Bonferroni’s method. Log2 transformed fold-change (Log2FC) was averaged in a similar fashion. A collective significance score (Sig) proportional to combined p-values and average Log2FC was generated and scaled from 0-100 to assess the degree of pathway enrichment or depletion relative to the inclusive set.

**Supplementary Data 6: Immune genes over-expressed in SNF2 metastases.** Immune genes were extracted from the Hallmark signatures ‘inflammatory response’, ‘interferon alpha response’, and ‘interferon gamma response’, in addition to the custom gene sets ‘immune estimate’, ‘immune msc’, ‘immune response’, and ‘immune Th1’. Shown are differentially expressed genes in the comparison of SNF2 metastases to SNF1 and 3 metastases. Fold-change denotes ratio of SNF2 vs. SNF1+SNF3. P-value corrected for multiple comparisons using the Benjamini–Hochberg method.

**Supplementary Data 7: Significantly mutated genes determined by MutSigCV.** All variants that passed validation criteria in coding regions were categorized and tabulated to create an overall mutation type summary for each gene. n_syn = number of synonymous mutations; n_mis = number of missense mutations; n_lof = number of loss-of-function mutations; n_splice = number of splice junction mutations; n_indels_mis = number of inserts/deletions causing missense mutations; n_indels_lof = number of insertions/deletions causing loss-of-function mutations; num_unique = number of unique instances of a point mutation seen. MutSigCV v1.2 determined the probability of base level mutations within specific gene-level contexts given overall mutation rate, ratio of synonymous to non-synonymous mutation types, and other gene-levels factors including estimates of expression, replication rate, and chromatin state^21^. Raw P-values indicate the probability that the number of somatic mutations found within each gene is observed by chance with multiple testing corrections controlled by false discovery rate (FDR, q-value).

**Supplementary Data 8: Genomic alterations unique to each SNF subtype.** Differentially enriched mutations and gene-level copy number variations are presented. Analysis of gene-level copy number variations was performed for those genes identified by TCGA in primary colorectal cancers^25^. Overall, analyses were performed for genomic aberrations with at least 20% frequency in at least one SNF subtype. Statistical significance was determined using Fisher’s exact tests between each subtype versus the remaining two subtypes.
